# P-1117. An Open-label Phase 1 Study in Healthy Adult Male Participants to Investigate the Absorption, Metabolism, and Excretion of [^14^C]-Rifaquizinone Following a Single Intravenous Administration

**DOI:** 10.1093/ofid/ofae631.1304

**Published:** 2025-01-29

**Authors:** Mohamed Al-Ibrahim, Bradly Keck, Marie Croft, Stephen English, Eleanor Barton, Loewe Kasprenski, Huan Wang, Changlin Ai, Guozhu Geng, Jing Chen, Zhenkun Ma

**Affiliations:** Pharmaron Clinical Pharmacology Center (CPC), Baltimore, Maryland; Pharmaron Clinical Pharmacology Center (CPC), Baltimore, Maryland; Pharmaron ABS, Inc., Germantown, Maryland; Pharmaron ABS, Inc., Germantown, Maryland; Pharmaron UK Ltd., Rushden, England, United Kingdom; Pharmaron Clinical Pharmacology Center (CPC), Baltimore, Maryland; TenNor Therapeutics (Suzhou) Ltd, Suzhou, Jiangsu, China (People's Republic); TenNor Therapeutics (Suzhou) Ltd, Suzhou, Jiangsu, China (People's Republic); TenNor Therapeutics (Suzhou) Ltd, Suzhou, Jiangsu, China (People's Republic); TenNor Therapeutics (Suzhou) Ltd, Suzhou, Jiangsu, China (People's Republic); TenNor Therapeutics, Suzhou Industrial Park, Jiangsu, China (People's Republic)

## Abstract

**Background:**

Rifaquizinone (RFQ, TNP-2092) is a novel multitargeting drug conjugate in development for the treatment of serious or life-threatening bacterial infections including those caused by gram-positive pathogens that have developed or acquired resistance to commonly used antibiotics and those associated with medical devices. RFQ exerts its antibacterial activity by inhibiting RNA polymerase, DNA gyrase, and topoisomerase IV. This study evaluated the absorption, metabolism, and excretion of RFQ following an intravenous (IV) administration in healthy participants.

**Figure:**
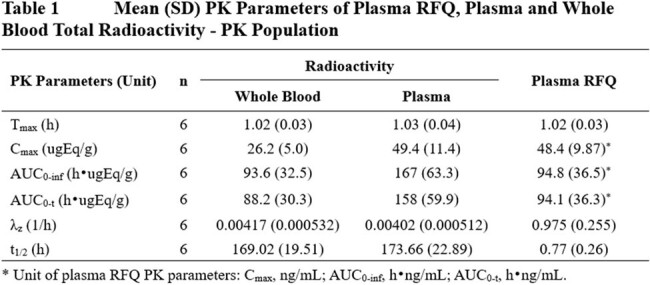

**Methods:**

This was an open-label phase 1 study (NCT05074134) conducted at a single site in the USA. Eligible adult male participants were administered a single dose of 300 mg/3 µCi [^14^C]-RFQ following a standardized meal. Blood, urine, and feces were collected up to 336 hours after initiating IV dosing for pharmacokinetic (PK), radiolabel recovery and metabolite assessments.

**Results:**

A total of 7 participants were enrolled and five participants completed the study. Six participants were included in the PK analysis. The majority (96.9%) of the administered radioactivity was excreted within 120 hours of IV dosing. A mean cumulative mass balance recovery of 102% was achieved after 336 hours post-dose with 98.7% and 2.74% recovered in the feces and urine, respectively. The PK profiles of radioactivity in whole blood and plasma, and RFQ in plasma are summarized in Table 1. The ratio of mean AUC of radioactivity between whole blood and plasma was 0.568. RFQ was identified as the major radioactive component in pooled plasma and pooled feces, accounting for 56.1% and 61.7% of total radioactivity, respectively. The C-25 O-deacetylated RFQ was identified as the major metabolite, accounting for 11.6% of the total radioactivity in pooled feces. No single radioactive component present in urine accounted for greater than 10% of the administered dose.

**Conclusion:**

After a single IV dose of 300 mg/3 µCi [^14^C]-RFQ, 96.9% of the administered radioactivity was recovered within 120 hours. The majority (98.7%) radioactivity was excreted in the feces. RFQ was the major radioactive component in pooled plasma and feces.

**Disclosures:**

**Mohamed Al-Ibrahim, MB ChB**, TenNor Therapeutics (Suzhou) Ltd: Investigator **Bradly Keck, PhD**, TenNor Therapeutics (Suzhou) Ltd: Investigator **Marie Croft, PhD**, TenNor Therapeutics (Suzhou) Ltd: Investigator **Stephen English, BS**, TenNor Therapeutics (Suzhou) Ltd: Investigator **Eleanor Barton, Master**, TenNor Therapeutics (Suzhou) Ltd: Investigator **Loewe Kasprenski, MBA**, TenNor Therapeutics (Suzhou) Ltd: Investigator **Huan Wang, PhD**, TenNor Therapeutics (Suzhou) Ltd: Employee **Changlin Ai, Master of Medicine**, TenNor Therapeutics (Suzhou) Ltd: Employee **Guozhu Geng, MD**, TenNor Therapeutics (Suzhou) Ltd: Employee **Jing Chen, Master of Science**, TenNor Therapeutics (Suzhou) Ltd: Employee **Zhenkun Ma, PhD**, TenNor Therapeutics (Suzhou) Ltd: Employee

